# A mouse model of chronic idiopathic pulmonary fibrosis

**DOI:** 10.1002/phy2.249

**Published:** 2014-02-25

**Authors:** Nathachit Limjunyawong, Wayne Mitzner, Maureen R. Horton

**Affiliations:** 1 Department of Environmental Health Sciences, Program in Respiratory Biology and Lung Diseases, Johns Hopkins Bloomberg School of Public Health, Baltimore, Maryland21205, USA; 2 Department of Medicine, Johns Hopkins University School of Medicine, Baltimore, Maryland21205, USA

**Keywords:** Bleomycin, chronic idiopathic pulmonary fibrosis, diffusing capacity, idiopathic pulmonary fibrosis, pulmonary function, quantitative histology

## Abstract

Chronic idiopathic pulmonary fibrosis (IPF) is a progressive, fatal, and untreatable disease with unclear etiology. There are few models of this chronic pathology, and although delivery of bleomycin to induce acute lung injury is the most common animal model of pulmonary fibrosis, there is considerable uncertainty about whether this acute injury resolves in those animals that survive. In this report, we have systematically followed groups of mice for up to 6 months following a single insult of bleomycin. We assessed changes in lung function and pathology over this time course, with measurements of the diffusion capacity for carbon monoxide, lung mechanics, quantitative stereology, and collagen. Our results show that, while there is some repair over this extended time course, the injury in the lung never fully resolves. This persistent degree of fibrosis may have similarities to many features of human IPF. Thus, these chronic fibrotic changes in mouse lungs could be a useful model to evaluate potential therapeutic interventions to accelerate repair and possible treat this debilitating disease.

## Abstract (precis)

Figure shows the durability of the bleomycin induced fibrosis over 6 month.

## Introduction

The most common animal model of pulmonary fibrosis involves a single intratracheal or intranasal delivery of bleomycin to lung. This insult generally results in a dose‐dependent damage to the lung, characterized by inflammatory cell infiltrates, collagen deposition, and parenchymal consolidation. The lungs are generally studied 7–14 days following a single insult, and there is often an implicit assumption that if the mice do not die, the lungs will recover and the mice will return to normal. While this bleomycin model has some gross similarities to human idiopathic pulmonary fibrosis (IPF), there are several obvious and substantial differences (Thrall and Scalise 1995; Chua et al. 2005). Perhaps the most relevant one is that IPF is a chronic disease that develops over a long time span, whereas the conventional single insult bleomycin model is generally studied less than a month following an acute lung insult.

In the present study, we examined mice up to 6 months following this acute bleomycin injury to test whether this could serve as a better model for the chronic aspects of IPF. Our results show that, despite much evidence to the contrary in murine models (i.e., that the injury resolves over time) (Phan et al. 1981; Izbicki et al. 2002; Gharaee‐Kermani et al. 2005; Lawson et al. 2005), the mice we studied do not recover from the initial bleomycin injury, and that the chronic fibrosis that develops involves decreased lung volumes, increased lung stiffness, impaired gas exchange, and increased inflammatory cell profiles. This model thus may be a better way to test potential therapies that could ameliorate the presence or progression of IPF.

## Methods

### Animal and bleomycin challenge

All of the experimental procedures used in this study were approved by the Institutional Animal Care and Use Committee of the Johns Hopkins University (Baltimore, MD). Female C57BL/6J mice (8 week old) were purchased from Jackson Laboratory (Bar Harbor, ME). To induce pulmonary fibrosis, mice were anesthetized with 5% isoflurane and administered bleomycin (APP Pharmaceuticals, Schaumburg, IL) at a dose of 0.005 U/g mouse via intratracheal aspiration on day 0. Control animals received an equal volume of sterile PBS only. Mice were studied at 1, 3, and 6 months after bleomycin insult.

### Diffusion factor for carbon monoxide measurement

To assess overall functional changes in the lungs following bleomycin‐induced injury, measurement of the diffusion factor for carbon monoxide (DFco) was performed as described previously (Fallica et al. 2011). Briefly, mice were anesthetized with a mixture of ketamine (100 mg/kg)/xylazine (15 mg/kg) via intraperitoneal injection. Once sedated, a tracheostomy was performed, and an 18‐gauge cannula was inserted. Mouse lungs were quickly inflated with a 0.6 mL gas mixture (0.5% neon, 1% CO and balance air). After a 9 sec breath hold, 0.6 mL of gas was quickly withdrawed from the lung and diluted to 2 mL with room air. The neon and CO concentrations in the diluted air were measured by gas chromatography (INFICON, Model 3000A; Inficon Inc., East Syracuse, NY) to assess diffusion factor for carbon monoxide. The dilution to 2 mL was needed, since the gas chromatograph required a minimal sample size of ≈1 mL.

### Pulmonary mechanics

After DF_CO_ assessment, mice were connected to a flexiVent™ ventilator (Scireq, Montreal, QC, Canada) and ventilated with a tidal volume of 0.2 mL of 100% oxygen at a rate of 150 Hz. with a positive end‐expiratory pressure (PEEP) of 3 cmH_2_O. Mice were paralyzed with an i.p. injection of Succinylcholine (75 mg/kg), subjected to deep inspiration at 30 cmH_2_O for 5 sec and returned to normal ventilation for 1 min. Baseline measurements of respiratory system resistance (*R*rs), compliance (*C*rs) and elastance (*E*rs) were measured during a 2‐s breath hold with a 2.5‐Hz sinusoidal oscillation using the single‐compartment model (Ewart et al. 1995). The impedance of the respiratory system was also obtained using a constant phase model to provide measurements of airway resistance (*R*_aw_), tissue damping (*G*), and tissue elastance (*H*) (Hantos et al. 1992). Next, the ventilation was stopped, and the tracheal cannula was occluded for 4 min to degas the lungs by absorption atelectasis and to allow the heart to stop. Quasistatic pressure‐volume (P‐V) curves were performed as previously reported (Soutiere and Mitzner 2004). Changes in lung air volume were determined by measuring the displacement of a syringe with a linear displacement transformer attached to a syringe pump (model 55‐2226; Harvard Apparatus, Holliston, MA) after correcting for gas compression in the system. Airway pressure (Paw) and volume were recorded by a PowerLab digital data acquisition system (AD Instruments, Castle Hill, NSW, Australia). Total lung capacity (TLC) and residual volume (RV) of each mouse were assessed as the volume at 35 cmH_2_O and −10 cmH_2_O respectively. The slope of the deflation limb between 3 and 8 H_2_O from the P‐V loop was defined as the quasistatic compliance of the respiratory system (Cstat).

### Lung collection and processing

Following measurement of pulmonary mechanics, the chest wall was opened, the right mainstem bronchus was tied off with suture, and the right lobes of the lung were dissected free, snap frozen in liquid nitrogen and stored at −80°C for hydroxyproline assay. The left lobe was then inflated with buffered zinc formalin at a pressure of 30 cmH_2_O. After 10 min, the lung was tied off, and the fixed left lung volume was measured by the water displacement technique (Scherle 1970). The left lung was cut into three or four pieces transverse to the long axis and then embedded in paraffin. Five‐*μ*m‐thick sections were cut from each of the pieces, and the sections were stained either with hematoxylin and eosin (H&E) to assess lung architecture and inflammatory cells or with Masson's Trichrome stain kit (American MasterTech, Lodi, CA) to visualize collagen deposition.

With the common paraffin embedding procedure we have used here, there is always a substantial degree of tissue shrinkage (≈15% linear). This is even more complex in the lung, which is likely very heterogeneous in how this shrinkage in an inflated lung occurs. This heterogeneous shrinkage has never been analyzed in normal lungs, let alone those with a heterogeneous fibrotic pathology. Here we have implicitly assumed that there was a relatively similar degree of shrinkage in normal and fibrotic lungs, and with this assumption we have compared the differences between control and fibrotic lungs. Since the lung surface area is determined by the number of intercepts of a line segment with alveolar walls, unless there is differential shrinkage of the inflated regions of fibrotic and control lungs, the differences between control and fibrotic lungs should still be detectable.

### Quantitative histology

Three sections from the upper, middle, and lower regions of the left lung were randomly selected for quantification. Fifteen to twenty digital images per section were taken with a Nikon Eclipse 80i (Nikon, Tokyo, Japan) at ×20 magnification after selection by the systematic uniform random sampling method (Hsia et al. 2010). The images were analyzed with sampling grid lines using Nikon's NIS Elements software. The stereological parameters of mean chord length (*L*_m_), volume of parenchyma (*V*_par_) and fraction of parenchyma (*F*_par_) were calculated as previously described (Vasilescu et al. 2012; Schneider and Ochs 2013). Because of the extensive pathologic deposition of fibrous tissue in this study, we also defined a new parameter which we termed the “fraction of tissue” (F_tissue_), determined by the proportion of points that landed on all tissue including both alveolar septal tissue and deposited extracellular matrix. Total surface area (SA) of left lung was calculated from the *L*_m_ and fixed left lung volume (SA = 4 *V*/*L*_m_) (Weibel 1963).

### Collagen content

To estimate amount of collagen in the lung, the right lungs were used for a hydroxyproline assay (Sigma‐Aldrich, St. Louis, MO) according to the manufacturer's protocol. Briefly, the lungs were weighed, homogenized in sterile water and hydrolyzed in 12N HCl at 120°C for 3 h. The hydrolyzed samples were incubated with 4‐(Dimethylamino) benzaldehyde (DMAB) for 90 min at 60°C, and absorbance of oxidized hydroxyproline was determined at 560 nm. The amount of collagen was expressed in micrograms per milligram lung tissue.

### Statistical analysis

Unless otherwise noted, the results are expressed as means ± SEM (with *n* = 6–8 mice per group). Statistical analyses were performed using GraphPad Prism 5 software (LA Jolla, CA). The comparisons between bleomycin group and PBS control group were analyzed by an unpaired *t*‐test. A one‐way ANOVA, followed by post hoc Bonferroni tests with two‐tailed distribution was used for analyzing the data between different time points. A *P* value <0.05 was considered significant.

## Results

### Effects of bleomycin on murine lifespan and weight

**Figure 1.**
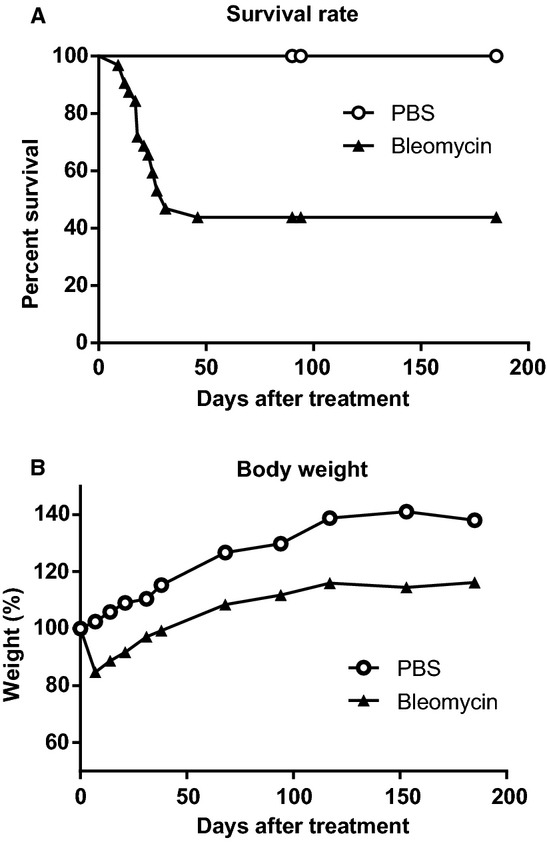
Survival rate and body weight. (A) Percentage of survival and (B) change in body weight of mice treated with bleomycin 0.005 U/g BW via aspiration. These data were observed to 6 months after instillation.

Figure 1A shows the survival of the control and bleomycin treated mice over the 6 month time interval. Over the first 30 days, slightly more than half of the treated mice failed to survive the single bleomycin insult. Beyond 30 days, however, there was no further attrition in those mice that survived. Figure 1B shows the changes in body weight in bleomycin treated and control mice. No weight loss was found in the PBS‐treated control group. However, weight loss is one of the traditional indicators of an acute bleomycin insult, and as expected, we found that mice that were given bleomycin demonstrated marked weight loss in the first week after challenge. The body weights plotted in this figure are only from those mice who survived the full 6 months. While these bleomycin treated mice show a growth rate similar to the control mice, over 6 months, there seems to be no trend to fully recover to reach the control body weights.

### Pulmonary function and lung mechanics

**Figure 2.**
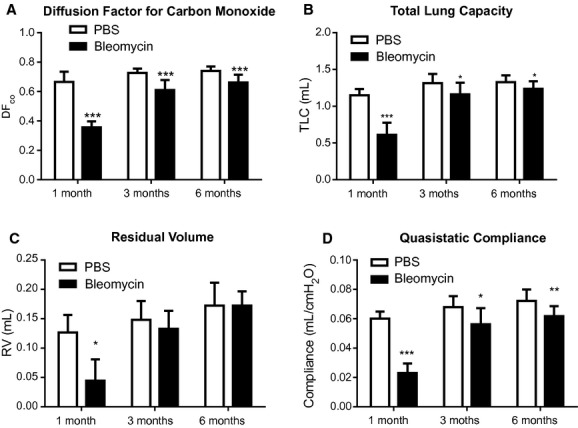
Measurements (mean ± SEM) of DF_CO_ and lung mechanics at different time points. (A) Diffusion factor for carbon monoxide (DF_CO_) was assessed by injecting air containing CO and Ne to the lung for 9 sec. While, (B) total lung capacity, (C) residual volume and (D) lung compliance were obtained from pressure‐volume curve generated after degassing the mice. *Open bar*: PBS control mice; *Solid bar*: bleomycin‐challenged mice (*P <**0.05, **P <**0.01, ***P <**0.001 in comparison to saline‐control mice at the same time point).

Lung gas exchange function in the control and fibrotic mice as assessed by the diffusing capacity is shown in Figure 2A. Control values of DF_CO_ (means ± SEM) at 1,3, and 6 months were 0.67 ± 0.031, 0.73 ± 0.011, and 0.74 ± 0.009, respectively, and there were no significant differences in DF_CO_ between these time points. In the bleomycin‐treated group, however, the DF_CO_ was significantly reduced to 0.36 ± 0.017 at 1 month post challenge, and this showed partial recovery at 3 and 6 months, where DF_CO_ was 0.61 ± 0.024 and 0.66 ± 0.016 respectively. These differences remained significantly different from the control mice at the same age (*P* < 0.05). These changes in gas exchange were supported by changes in lung mechanics as shown in Figure 2B–D. Bleomycin resulted in significant decreases in total lung capacity, residual volume and quasistatic compliance at 1 month. After this time, there was some partial recovery, but the total lung capacity and compliance in the bleomycin treated mice remained significantly different from controls. Residual volume, however, recovered to control levels. This recovery was also found in the dynamic mechanics measurements (*R*rs, *C*rs, *E*rs, *G,* and *H*), which only showed significant differences between treated and untreated mice only at 1 month (Table 1).

**Table 1.** Baseline values for the lung parameters: respiratory system resistance (Rrs), elastance (Ers), compliance (Crs), airway resistance (*R*_aw_), tissue damping (*G*), and tissue elastance (*H*) displayed by time points and groups.

Treatment	Rrs (cmH_2_O s/mL)	Crs (mL/cmH_2_O)	Ers (cmH_2_O/mL)	Raw (cmH_2_O.s/mL)	G (cmH_2_O/mL)	H (cmH_2_O/mL)
1 month						
****PBS	0.595 ± 0.021	0.040 ± 0.002	26.057 ± 1.662	0.338 ± 0.017	3.570 ± 0.218	25.439 ± 1.487
****Bleomycin	1.035 ± 0.097*	0.019 ± 0.002*	55.150 ± 5.591*	0.363 ± 0.016	7.411 ± 0.723*	51.699 ± 5.565*
3 months						
****PBS	0.598 ± 0.027	0.041 ± 0.002	24.446 ± 1.172	0.317 ± 0.025	3.488 ± 0.207	24.634 ± 1.853
****Bleomycin	0.668 ± 0.039	0.036 ± 0.003	28.440 ± 2.052	0.376 ± 0.021	4.088 ± 0.292	25.724 ± 1.716
6 months						
****PBS	0.590 ± 0.042	0.045 ± 0.002	22.524 ± 1.023	0.342 ± 0.040	3.767 ± 0.248	21.471 ± 1.539
****Bleomycin	0.674 ± 0.025	0.040 ± 0.001	25.170 ± 0.760	0.374 ± 0.024	4.394 ± 0.171	22.690 ± 0.987

Results are expressed as mean ± SEM.

* *P *<**0.01 for each time points compared to PBS‐treated mice.

### Histology and quantitative analysis

**Figure 3.**
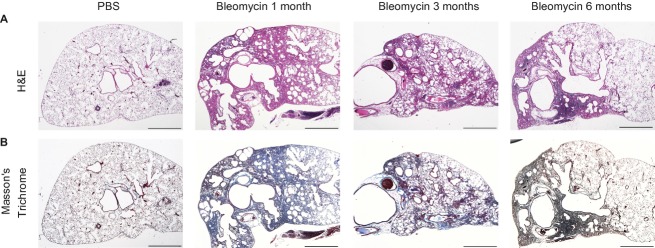
Representative photomicrographs of bleomycin‐ and saline‐treated lungs. (A) Hematoxylin and eosin stained. (B) Masson's Trichrome stained (buffered zinc formalin fixed, paraffin blocked, 5‐*μ*m‐thick sectioned, original magnification: 20X). Bar = 1000 *μ*m.

Figure 3 shows low magnification images of typical bleomycin‐treated lungs at the 3 post‐bleomycin time points. The control lungs (not shown) at these later time points would all look identical to the PBS controls at time zero. These images show very extensive structural changes in the lungs at 1 month, but the extent of these areas of consolidation lessens over time. However, even at 6 months, there are still regions that are grossly consolidated. Although not easily apparent from these low power images, in response to bleomycin‐induced lung injury, a large increase in inflammatory cells was observed in the bleomycin‐treated lungs at 1 month. This cellular infiltrate, however, was largely gone at 3 and 6 months.

**Figure 4.**
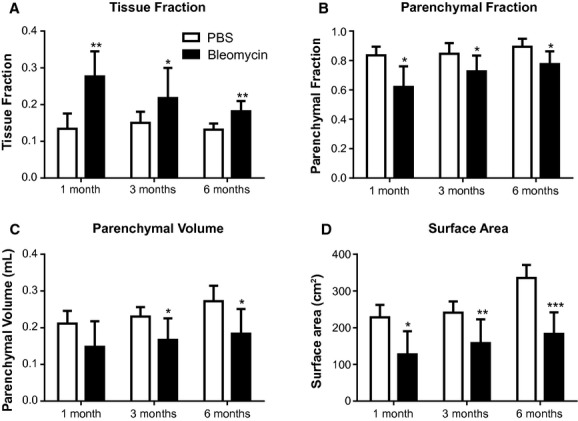
Quantitative histology in bleomycin‐ and saline‐treated mice at different time points. (A) tissue fraction, (B) parenchymal fraction, (C) parenchymal volume, and (D) surface area obtained by stereological analysis on histological sections. Data displayed as the mean ± SEM. *Open bar*: PBS control mice; *Solid bar*: bleomycin‐challenged mice (*P < 0.05, **P <**0.01, ***P <**0.001 for PBS vs. bleomycin group within each time point).

Figure 4A shows the tissue fraction in the lungs in control and bleomycin treated mice at the 3 time points. There were significant increases in the tissue fraction at all time points with a trend to lessen over time. This finding was mirrored in the parenchymal fraction (Figure 4B). Figure 4C shows the parenchymal volume of the two groups. There was a slight trend for the parenchymal volume in control mice to increase over time, and this was similar in the bleomycin treated mice. There was no tendency for this difference to lessen over time, and this stable difference mirrors the lack of recovery of the body weight. A similar phenomenon is also apparent in the parenchymal surface area (Figure 4D) which shows a significant loss of surface area that demonstrates no tendency toward recovery.

### Collagen deposition

**Figure 5.**
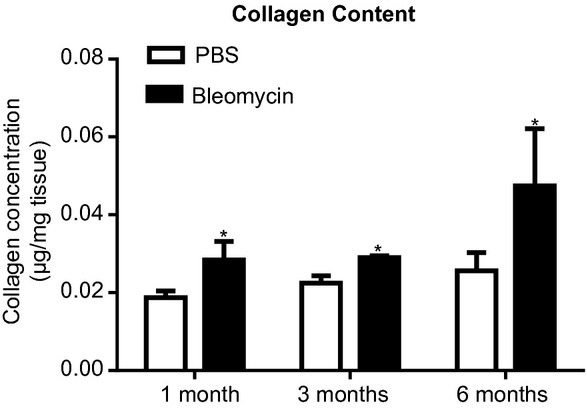
Hydroxyproline content in lung obtained from bleomycin‐ and saline‐treated mice. Time course for changes in collagen content in right lung homogenate samples measured by hydroxyproline assay. *Open bar*: PBS control mice; *Solid bar*: bleomycin‐challenged mice (*P <**0.05 compared with saline‐control mice at the same time point).

We used the hydroxyproline assay to quantify the changes seen in the collagen content in the lung histological sections. This global quantifying assay showed a similar result to that found with the quantitative histology (Fig. 5). There was an increase in collagen deposition in the bleomycin group at 1 month following bleomycin insult. This increased collagen in the bleomycin group remained significantly higher than controls at the 3 and 6 month time points.

## Discussion

In this study, we show that in the mouse, a single acute instillation of bleomycin leads to a fibrotic lung with chronic changes in lung structure and function, lung mechanics, lung volume, and body weight, consistent with what is seen in humans. We are unaware of any mouse models that have examined these chronic changes that develop and persist after a single bleomycin instillation, and most investigators study only the acute changes that occur within a few weeks involving a relatively rapid repair of the lung injury. While such a short time course could allow one to study drugs that might minimize the acute injury and repair following bleomycin, one would need a much longer time course to investigate drugs that might result in repair of fibrotic tissue. The model we have described here could be used to such ends.

To put our model in perspective, we consider its utility in the context of other relevant animal models of IPF. As there have been several recent general reviews of such models (Chua et al. 2005; Moeller et al. 2008; Moore and Hogaboam 2008; Mouratis and Aidinis 2011; Moore et al. 2013), here we will only focus on those models that are most closely related to the chronic model we have described here. These more relevant models are those that involve the administration of repeated doses of bleomycin over time either delivered systemically (intraperitoneal [IP], Adamson and Bowden 1974; Ezzie et al. 2011, or subcutaneous, Harrison and Lazo 1987; Rydell‐Tormanen et al. 2012), or intratracheally (Chung et al. 2003; Degryse et al. 2010).

Repeated doses of intratracheal (IT) bleomycin were used to establish persistent fibrosis (Chung et al. 2003; Degryse et al. 2010). However, this is a much more cumbersome model to use with a large cohort. The model we have described uses only a single insult that also results in persistent fibrosis, so there would seem little reason to go to the extra trouble of repeatedly anesthetizing many mice to give the successive IT challenges.

It is worth commenting here on the relevance of the bleomycin model in general. Some have considered bleomycin more as a model of acute lung injury followed by scar formation, but not a model of IPF, because IPF is not only thought to result from repetitive minor epithelial injuries coupled with impaired wound healing, but is also characterized by spatial and temporal heterogeneity. The remodeling of an existing scar in IPF is a progressive process with little evidence of even partial resolution. Hence, active sites with injury and profibrotic remodeling may coexist with endstage fibrosis in the same lung. Indeed, the relevance of the bleomycin model to human IPF has been questioned for decades. In general the study of human pulmonary fibrosis has been complicated by the unknown etiology, variable natural history, advanced staged disease at presentation and the insidious progression of fibrosis over years and even decades. The limitation of all the animal models of fibrosis is that they attempt to reproduce years of human damage due to unknown insults in a brief period time after a single insult (Chua et al. 2005; Moeller et al. 2008; Moore and Hogaboam 2008). The bleomycin model of in vivo lung injury is a well‐described model allowing investigation of the elaborate pathways resulting in chronic inflammation and fibrosis (Bowden 1984). Although this model has been similarly criticized for its speed and durability of fibrosis, it remains a robust, reproducible model of general lung fibrosis (Chua et al. 2005; Moeller et al. 2008; Moore and Hogaboam 2008). Thus, although no animal model of lung fibrosis is perfect, the bleomycin model is a well‐established, well‐studied, reproducible model of general lung fibrosis that can be interrogated to reveal potential mechanisms underlying human fibrotic lung disorders (Chua et al. 2005; Moeller et al. 2008; Moore and Hogaboam 2008; Wilson et al. 2010). In our case, even if the injury is recovering very slowly, the fact that the injury persists over 30% of the mouse's life span makes it a durable model in which it is possible to test therapies that can hasten that resolution.

Bleomycin has also been given systemically in small doses over extended time periods for up to several weeks. The delivery can be done either IP or subcutaneously, and, unlike the repeated IT delivery, it does not require anesthesia. However, because there is a cumulative effect of such repeated doses, it is not clear that the mice ever reach a stable fibrotic condition. One could argue that this model may better mimic the human condition if there was a continuous stimulation in humans that leads to the observed chronic pathology. However, such a situation has not been shown in humans, so this repeated dose model has the same relevance as the single dose model. The single dose model as described here, however, is a much simpler model to implement.

One of the other problems with only examining the acute responses over the first month following a single bleomycin insult is that the responses to antifibrotic or anti‐inflammatory drugs is very sensitive to the timing and duration of treatment after the initial insult (Chaudhary et al. 2006). The availability of a stable chronic injury allows investigators to test new potential therapies to see if any can possibly ameliorate or even reverse the pathology. The issues with timing and duration intrinsic to prevention of the injury in the acute model become secondary in our chronic model.

One thing that we emphasized in the present work is the measurement of pulmonary function. While such measurements have long been available in animal models, functional measurements have been the exception rather than the rule in animal models of fibrosis. It is not clear why this has been, since most measurements of lung function are relatively straightforward to make. We found that the chronic fibrosis in our model showed decreased diffusing capacity, decreased lung capacity, and decreased lung compliance. In addition to these functional measurements, the quantitative histology also showed a significant loss of functional parenchymal tissue, consistent with the reduced diffusing capacity. These changes are typical of those seen in human IPF, further supporting the relevance of this mouse model. While lung volumes and compliance are not generally done in humans, the diffusing capacity provides a functional metric that not only is easily done in humans, but also can be used to follow changes in the level of fibrosis over time or with experimental therapies.

Finally, it is worth noting that although the use of bleomycin to induce a model of chronic fibrosis has not been done in the mouse, it was originally shown in the hamster (Snider et al. 1978; Goldstein et al. 1979) and rat (Borzone et al. 2001). Furthermore, pulmonary function measurements were also done in this hamster model. For reasons that are not entirely clear, there were no subsequent mechanistic studies following these initial hamster and rat studies. The fact that many responses in the mouse model are similar to those found in the hamster supports the validity of our present findings. The rat was only studied at one time point (4 months) after the initial bleomycin, and the authors found no change in lung compliance at that time despite there being considerable peribronchiolar inflammation. Thus, it seems that the rat may have a qualitatively different response than either the hamster or the mouse. Given the modern ability to manipulate genes and signaling pathways in the mouse, there should be greater opportunity to use the mouse for more mechanistic and possible therapeutic insights.

In summary, we have shown that, after a single intratracheal dose of bleomycin, the mouse lung does not fully recover. The lung shows a chronic injury that is consistent with many features of IPF in humans. This chronic model has the capacity to allow testing of new compounds that could slow or reverse the damaged tissue in a fibrotic lung.

## Conflict of Interest

No conflicts of interest, financial or otherwise, are declared by the author(s).
